# A Dive Into Oblivion: A Case of Transient Global Amnesia

**DOI:** 10.7759/cureus.59603

**Published:** 2024-05-03

**Authors:** Inês Martins, Tiago Araújo, Inês Madeira, João Frederico Ribeiro, Ana Fernandes

**Affiliations:** 1 Department of Critical Care Medicine, Unidade Local de Saúde do Litoral Alentejano, EPE, Santiago do Cacém, PRT

**Keywords:** anterograde amnesia, retrograde amnesia, cold water immersion, amnesia, transient global amnesia

## Abstract

Transient global amnesia (TGA) is an uncommon neurologic disorder that consists of a sudden and temporary loss of memory, both present and past. Its causes and risk factors are not well known.

We describe a case of a 58-year-old woman who was brought to the emergency department (ED) with sudden onset loss of memory and disorientation after a dive in the ocean. She presented memory deficits with incapacity to retain new memories and amnesia for the previous 24 hours. All exams ordered were normal, including computed tomography of the brain and laboratory analysis. After six hours of close monitoring in the ED, she gradually started to retain short-term memories and was discharged after 48 hours with no memory or other deficits.

The diagnosis of TGA was made based on the clinical presentation and the patient’s rapid improvement. Follow-up neurology consultation and further testing did not demonstrate any evidence to exclude this diagnosis.

Further research is needed on this topic to allow the identification of risk factors and causes to prevent it.

## Introduction

Transient global amnesia (TGA) is a neurological disorder characterized by sudden onset and usually self-limited complete anterograde amnesia, frequently associated with retrograde amnesia. These memory changes can last from several hours to days [1,2].

The diagnosis of TGA can only be made in the absence of head trauma or seizures in the 24 hours previous to the ensuing symptoms [2]. The estimated incidence of TGA is between 3-8/100,000 population per year, and its etiology and risk factors are not entirely known [1-4].

The only cognitive function affected is memory, both anterograde and retrograde, while other cognitive functions remain intact. The patient’s ability to recognize previously known family and friends is unimpaired; however, it becomes impossible to create new short-term memories of people, places, and events. Also, retrograde amnesia can extend to nearly 24 hours before the trigger event [2].

TGA differential diagnosis is extensive and includes other transient memory disturbs, epileptiform or concussional transient amnesia, and several other intracranial injuries (e.g., astrocytomas) or aura from migraines [2]. The prognosis is usually very good with complete recovery after 24 hours in most cases [3,4].

## Case presentation

A 58-year-old woman with a history of depression, medicated at home with paroxetine (20 mg once daily), trazodone (150 mg once daily), and escitalopram (10 mg once daily), was brought to the emergency department (ED) due to sudden onset disorientation after a dive in the sea, with total body immersion in cold shallow water, using no diving equipment. At the time of the event, she was on a weekend vacation with a friend. The friend denied any head trauma or loss of consciousness as well as other symptoms, before or after the dive. After the patient emerged from the water, she was disoriented in space and time and initiated a repetitive speech, constantly asking where she was and how she got there. According to the friend, she also could not remember the previous 24 hours, so the last thing she remembered was the car ride to where they were staying on their vacation.

On physical examination at the ED, the patient presented repetitive speech, frequently asking “Where am I?” or “What happened?”. At the same time, her ability to form short-term memories was impaired. This was evident because in the first hours of her stay at the ED, she was impaired to the point of not recognizing the doctor after repeated evaluations. She could remember her full name and personal information but, when asked, the last thing she could remember was her car ride for the weekend getaway. She was also unable to correctly identify where she was and presented amnesia to the moment she dove into the sea. Further neurologic examination, including a complete neurologic physical exam, was normal, as were all vital signs.

Additional testing was performed to exclude other causes for the sudden memory impairment. Laboratory analysis showed no changes in inflammatory markers, thyroid function, cardiac biomarkers, and coagulation tests. Also, urine testing showed no signs of infection, and the toxicology screen was negative for opioids, cannabinoids, cocaine, tricyclic antidepressants, and benzodiazepines. Contrast-enhanced computed tomography (CT) was also normal. Noncontrast brain magnetic resonance imaging with diffusion-weighted imaging (MRI-DWI) was not performed due to lack of availability. Lumbar puncture was not deemed needed because the patient presented no clinical signs of central nervous system infection.

She remained in the ED for observation, and about three to four hours after admission, she gradually became able to remember the ED healthcare team and started retaining higher volumes of information given to her, initially by periods of 10 to 15 minutes, and then progressively longer. During these first hours, there was no improvement in the 24-hour retrograde amnesia.

The absence of changes in all of the performed exams in conjunction with the findings on physical examination made, the diagnosis of TGA very likely. Neurology consultation was required and 24 hours after admission, the patient was discharged home with an outpatient neurology appointment.

In subsequent follow-up appointments, brain magnetic resonance imaging (MRI) was performed, revealing no changes in white or grey matter and normal size and permeability of the ventricular and vascular systems. An electroencephalogram (EEG) showed normal results without epileptiform discharges. The patient is still accompanied at the neurology outpatient clinic, with no record of any neurological impairment.

## Discussion

Although there is not much information regarding the etiology of TGA, it is known that it is more common between the fifth and seventh decades of life, and is usually associated with a trigger, like extenuating physical activity, cold water immersion, receiving bad news, sexual intercourse, or situations associated high levels of stress [1,2,4]. There are several theories that propose vascular phenomena, migraine, or epilepsy in the genesis of TGA but they are yet to be proven [1].

The risk factors for TGA are not properly identified, but in later studies, there seems to exist a positive association with migraines [1,2]. This patient fits the age range in which TGA is more common and the episode occurred after cold water immersion, which is one of the well-established triggers.

The presenting symptoms of TGA include sudden onset anterograde amnesia from the inciting trigger, but sometimes also retrograde amnesia [1,2]. Repetitive speech is common in these patients, constantly asking questions like “Where am I?”, “How did I get here?”, or “What time is it?”, but not being able to remember the answers that were given to them [2]. The diagnosis is clinical, combining the previous symptoms with no loss of personal identity, absence of head trauma, and focal neurological signs. The entirety of symptoms must last from a minimum of one hour up to a maximum of 24 hours after the trigger event [1,2,4].

The criteria defined by Hodges and Warlow in 1990 perfectly summarize the clinical features of TGA: abrupt onset memory deficit that must be witnessed; obvious anterograde amnesia with repetitive questions; mild retrograde amnesia and difficulty on executive function, without cognitive impairment; no occurrence of head trauma or change in consciousness during the event; no loss of personal identity; no focal neurologic deficits after the event; total duration of symptoms between one hour to 24 hours [5]. In the case we describe, we could easily identify the anterograde memory impairment due to the typical repeated questions and the inability to remember the attending healthcare personnel in the absence of any other neurologic deficit. Additionally, the presence of retrograde amnesia extending 24 hours prior to the trigger is also a common finding.

By definition - hence the term transient - these episodes resolve in several hours to a few days. However, some recent studies defend that it should not last more than 24 hours [1,2,4]. In our patient, the anterograde amnesia began resolving progressively, starting at three to four hours post event while the retrograde amnesia took a while longer but was completely resolved by the end of the first 24 hours.

Some conditions can present with memory impairment similar to TGA, like postconcussion syndrome, seizures, and intracranial injuries [2]. Transient epileptic amnesia (TEA) is also a differential diagnosis to consider. TEA is a form of focal epilepsy originating in the temporal cortex with presenting clinical features very similar to TGA [6]. The key difference is that TEA can present as recurrent episodes of TGA and has other manifestations besides memory loss, for example, language disorders [6].

Although there is no need for additional testing when the presentation is typical, it is not unusual to perform complementary exams to exclude other causes, like blood analysis, including a toxicology screen, head CT, brain MRI, and EEG. In cases of atypical presentations, these exams should be performed. Noncontrast brain MRI-DWI may show typical punctate lesions of the hippocampus, seen in up to 75% of patients with TGA. However, the absence of said lesions does not exclude TGA, as in some patients they can be seen outside of the hippocampus but also because they are most likely to be seen 24-72 hours after the event, and be absent in the hyperacute phase [4].

The treatment of TGA is directed to symptom control, especially the anxiety that memory loss triggers [1,2].

The prognosis of TGA is usually very good, although there might be some recurrent episodes as described by Hernández et al. in a meta-analysis published in 2022, which found that recurrence usually happens in one in every eight cases and is associated with current migraine, depression, or sexual intercourse before the event [4,7]. Because of this risk, it is important to maintain follow-up and make sure no new episodes ensue and no sequela develops.

## Conclusions

TGA is a relatively uncommon condition with no clearly identifiable risk or predisposing factors. Several events are explicitly linked to episodes of TGA but the mechanism through which it occurs is not well known. In this case, the diagnosis was made based on presenting symptoms and clinical examination and was confirmed by the self-resolution within 24 hours.

Further studies and research are needed on this topic to identify risk factors and causes so that the population at risk is recognizable, and these episodes can be prevented.
